# Glyoxal Formation and Its Role in Endogenous Oxalate Synthesis

**DOI:** 10.1155/2012/819202

**Published:** 2012-04-08

**Authors:** Jessica N. Lange, Kyle D. Wood, John Knight, Dean G. Assimos, Ross P. Holmes

**Affiliations:** Department of Urology, Wake Forest University Medical Center, Medical Center Boulevard, Winston-Salem, NC 27157, USA

## Abstract

Calcium oxalate kidney stones are a common condition affecting many people in the United States. The concentration of oxalate in urine is a major risk factor for stone formation. There is evidence that glyoxal metabolism may be an important contributor to urinary oxalate excretion. Endogenous sources of glyoxal include the catabolism of carbohydrates, proteins, and fats. Here, we review all the known sources of glyoxal as well as its relationship to oxalate synthesis and crystal formation.

## 1. Introduction

Endogenous oxalate synthesis makes an important contribution to the amount of oxalate excreted in urine and hence the development of calcium oxalate kidney stones, a painful condition that afflicts 10% of the adult Caucasian male population in the United States. The biochemical pathways involved in oxalate production are poorly understood despite decades of research. However, it is clear that glyoxylate is the major precursor of oxalate [1]. The metabolism of a number of substances has been proposed as a source of glyoxylate including glycine, phenylalanine, tryptophan, hydroxyproline, glucose, fructose, pentose sugars, ethanolamine, and glycolate. Our research to date indicates that of these potential sources, hydroxyproline makes a modest contribution, 5–10%, and the majority of others provide negligible amounts [2–4]. We have found, however, by incubating a variety of 2 carbon molecules with hepatocytes, that glyoxal is a prominent source of glyoxylate [3]. We hypothesize that glyoxal is one of the most important sources of endogenous oxalate synthesis in humans and possibly other organisms and is one that has been overlooked to date. Mounting evidence is also suggesting that both glyoxal production and oxalate synthesis can be associated with oxidative stress.

## 2. Glyoxal

Glyoxal (O=CH–CH=O) is an *α*-oxoaldehyde, and it is often grouped with two similar *α*-oxoaldehydes, methylglyoxal, and 3-deoxyglucosone. All three compounds are products of various metabolic and oxidative reactions and are capable of causing cellular damage and apoptosis [5]. They are also involved in the formation of advanced glycation end-products (AGEs) which have been linked to long-term sequela of chronic diseases such as diabetic retinopathy, neuropathy, and nephropathy [6]. Glyoxal is primarily detoxified by the glyoxalase system present in the cells of bacteria, protozoa, fungi, plants, animals, and humans [7]. However, it has been suggested that several other enzymes are capable of detoxifying glyoxal, including aldehyde dehydrogenase (ALDH) which can oxidize glyoxal to glyoxylate [5, 6].

## 3. Sources of Glyoxal

Glyoxal originates from both exogenous and endogenous pathways. Exogenous sources are primarily dietary, and these include beer, wine, tea, coffee, yogurt, bread, rice, soybean paste, soy sauce, and oil [8]. Foods that have been fermented, roasted, baked, or fried are also a rich source of glyoxal. Environmental sources of glyoxal include cigarette smoke, smoke from residential log fires, vehicle exhaust, smog, fog, and some household cleaning products. It may also be introduced into the air during combustion of various materials. It has been found in samples of soil, groundwater, seawater, and sediment. Whether glyoxal from these sources is absorbed into the body through the gut, lungs, or skin to significantly affect glyoxal levels in tissues is not known with certainty.

**Figure 1:**
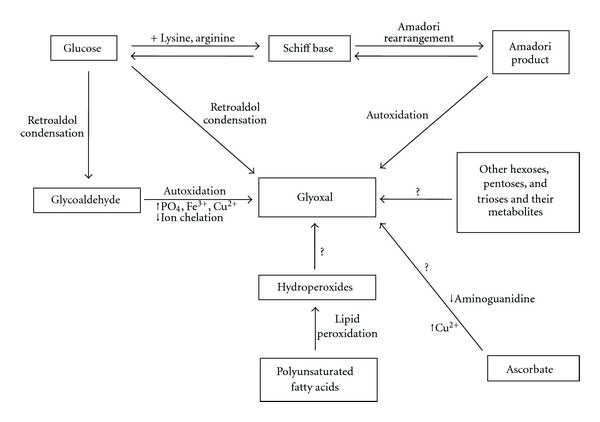
Sources of glyoxal.

In addition to the many exogenous sources of glyoxal, it is endogenously produced via autoxidation of carbohydrates and ascorbate, degradation of glycated proteins and lipid peroxidation [9]. Glyoxal can be formed through various pathways as illustrated in Figure 1. It is created directly from glucose via retroaldol condensation, and it is formed indirectly from glucose via a glycoaldehyde intermediate that undergoes autoxidation. The autoxidation reaction is promoted by the presence of phosphate buffer and trace metal ions (Fe^3+^ and Cu^2+^) in solution. The latter was indirectly demonstrated by a reduction of glycoaldehyde autoxidation by the presence of the trace metal ion chelator diethylenetriaminepentaacetic acid [10].

Another way in which glucose can be converted to glyoxal is via a Schiff base intermediate [10]. A Schiff base is a functional group that contains a carbon-nitrogen double bond and is formed from the acyclic form of glucose reversibly reacting with an amino acid (typically lysine or arginine) [11]. Once the Schiff base forms, it exists primarily in the cyclic form, but it can exist in an acyclic form that reversibly rearranges through an Amadori rearrangement to an Amadori product. The Amadori product can either be oxidized by a metal catalyst to form an AGE or undergo autoxidation and produce glyoxal [10]. It is through this mechanism that AGEs and fructosamine, an Amadori product, contribute to glyoxal production [6, 10].

Manini et al. have demonstrated that glyoxal cannot only be formed from glucose but from many other carbohydrates as well, including galactose, mannose, fructose, ribose, arabinose, ribulose, glyceraldehyde, acetone, adenosine, mannitol, and glycerol [12]. The mechanism by which glyoxal is formed from carbohydrates, including identification of intermediates, has been extensively studied by Hofmann et al. [13]. Ascorbate will also spontaneously hydrolyze into glyoxal via an unknown mechanism [14].

Although sugars are often regarded as the main source, lipid peroxidation is also a potentially important source of glyoxal. Three general steps occur during autoxidation of lipid molecules: initiation, propagation, and termination. In this case, initiation occurs when polyunsaturated fatty acids such as linoleic and linolenic acid undergo nonenzymatic lipid peroxidation. The resultant product is a hydroperoxide which then degrades into a variety of oxidation products, including glyoxal [15]. Spiteller et al. have suggested that such aldehydes are formed by a second dioxygenation of the primary hydroperoxides formed [16]. Glyoxal has been identified among the autooxidation products of linoleic and linolenic acids [17, 18] and has been detected in ischemic porcine heart tissue [19]. Clearly, the formation of glyoxal associated with the peroxidation that occurs in a number of disease states warrants further investigation.

## 4. Metabolism

The bulk of the glyoxal formed in tissues is converted to glycolate by the glyoxalase system (consisting of glyoxalase I and glyoxalase II) located in the cytosol of all cells [5]. Glyoxalase I also exists in the endoplasmic reticulum of cells, while glyoxalase II can additionally be found in the mitochondria. The activity of glyoxalase I is greatest in human pancreas, lung, kidney, and brain tissue and lowest in adipose and liver tissue. Interestingly, the glyoxalase I activity of fetal tissues is three times higher than in adult tissues [7]. It is also possible that glyoxal could be converted to glycolate by free radical initiated reactions, but such a nonenzymatic pathway has not yet been identified in biological systems.

Glyoxal conversion to glycolate requires glutathione (GSH). In situations where GSH is depleted, as might occur with oxidative stress, other enzymes such as aldehyde reductase, aldose reductase, carbonyl reductase, aldehyde dehydrogenase, and 2-oxoaldehyde dehydrogenase may be involved in the metabolism of glyoxal [5, 6, 20]. The presence of sorbinil, an aldose reductase inhibitor, has been found to inhibit metabolism of glyoxal, leading to high intracellular concentrations and eventual cell death [6]. Elevated intracellular glyoxal levels may inhibit aldehyde reductase, glutathione reductase, and NADPH-producing enzymes. In addition, high glyoxal concentrations produce reactive oxygen species (ROS) and formaldehyde, they increase cell susceptibility to hydrogen peroxide, and they disrupt the mitochondrial membrane potential, further showcasing its toxic effects [6, 21].

Certain substances have been found to increase the rate of glyoxal metabolism and are thus cytoprotective. These include antioxidants (e.g., butylated hydroxyanisole), iron chelators (e.g., desferoxamine), and NADH generators (e.g., xylitol and sorbitol) [6, 21]. The decrease in cytotoxicity in the presence of the antioxidant reinforces the hypothesis that ROS produced from lipid peroxidation may contribute to the toxic effects of glyoxal [6]. In addition, Shangari et al. found that the extent of cellular damage caused by glyoxal accumulation in hepatocytes could be decreased by the use of glyoxal “traps,” including d-penicillamine, aminoguanidine, arginine, cysteine, and pyridoximine. They also prevented GSH depletion and lipid peroxidation from occurring. Thiamine deficiency also increases hepatocytes susceptibility to glyoxal toxicity [21]. These studies demonstrate that glyoxal metabolism and its toxicity are complex, and further research is required to resolve the mechanisms involved.

## 5. Relationship between Glyoxal and Oxalate

**Figure 2:**
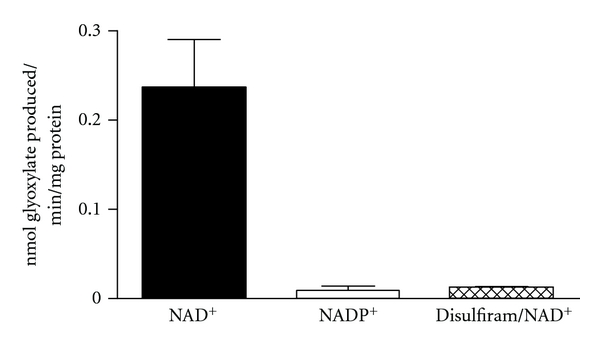
Conversion of glyoxal to glyoxylate in liver tissue homogenates. Incubations contained 0.2 mM glyoxal, 50 mM TRIS pH 9.0, 100 mM KCl, 1 mM EDTA, 1 mg human liver lysate, 10 minutes, 37°C. Reaction additions: 1 mM NAD^+^, 1 mM NADP^+^, 1 mM disulfiram. Glyoxylate was measured in perchloric acid extracts by reversed phase HPLC after derivatization with 10 mM phenylhydrazine.

**Figure 3:**
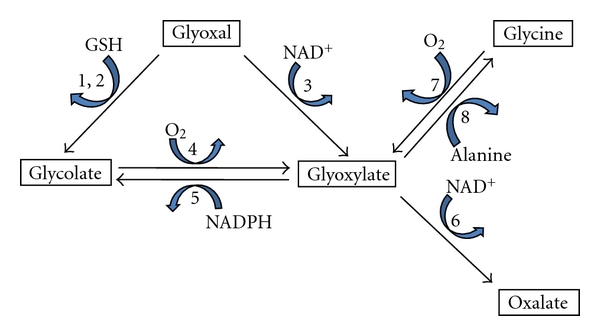
Potential mechanisms for oxalate synthesis from glyoxal. Enzymes involved include (1) glyoxylase I, (2) glyoxylase II, (3) aldehyde dehydrogenase, (4) glycolate oxidase, (5) glyoxylate reductase, (6) lactate dehydrogenase, (7) D-amino acid oxidase, and (8) alanine : glyoxylate aminotransferase.

There is both inferential and direct evidence that glyoxal is involved in endogenous oxalate synthesis. Inferential evidence is provided by studies of diabetics. They have higher plasma glyoxal levels than controls (perhaps due to increased lipid peroxidation and protein glycation) as well as increased urinary oxalate excretion [22–24]. Diabetics also have a higher risk of stone formation [25]. Direct evidence was obtained in studies with HepG2 cells, a hepatoma-derived cell line. When they were incubated with glyoxal, oxalate was generated [3]. Incubation of liver homogenates with glyoxal further revealed that glyoxal can be converted to glyoxylate, the major precursor of oxalate, by an NAD^+^-dependent reaction (Figure 2). The inhibition of the reaction by disulfiram suggests that the reaction is being catalyzed by an aldehyde dehydrogenase. The possible metabolism associated with the conversion of glyoxal to oxalate is depicted in Figure 3.

 This proposed pathway for oxalate generation has several repercussions for identifying the pathways that contribute to endogenous oxalate synthesis and developing strategies to modify calcium oxalate stone formation. Sources of glyoxal should be identified, steps involved in the conversion of glyoxal to oxalate determined, the role of oxidative stress and inflammation studied, and the role of antioxidant therapies in decreasing oxalate synthesis examined. Furthermore, the contribution of individual tissues to whole body oxalate production in various metabolic states warrants clarification. An important question is whether an increased endogenous oxalate synthesis is an index of whole body peroxidation. Such analyses will, however, require a careful separation of the oxalate derived from this source and that derived from the diet. As an increased glycolate synthesis should occur in association with glyoxal formation, urinary glycolate excretion may give a “cleaner” view of whole body peroxidation. In this regard, it has been previously noted that, in some idiopathic calcium oxalate stone formers, there is an apparent association between elevated excretions of both oxalate and glycolate [26, 27].

## 6. Calcium Oxalate in Tissues

The accumulation of calcium oxalate deposits in tissues is known as oxalosis. Oxalosis can occur via both hereditary (i.e., primary hyperoxalurias) and other mechanisms. The accumulation of small amounts of calcium oxalate crystals in the absence of aberrant oxalate metabolism has been termed dystrophic oxalosis [28]. This has been reported in a number of tissues including arterial atherosclerotic plaques, myocardium, lymph nodes, testis, thyroid, breast, ocular tissues, and kidneys with acquired cystic disease [28, 29]. In the study of calcium oxalate deposits in coronary artery atherosclerotic plaques, the 4 patients all had chronic diseases that can increase oxidative stress levels, including AIDS, hepatitis C, sepsis, ischemic heart disease, and cancer [28]. Calcium oxalate crystals were also found in myocardium, lymph nodes, testis, and thyroid tissues [28]. Oxalate crystals have frequently been observed in thyroid and breast tissue and are typically linked to benign disease more than malignant [30, 31]. Calcium oxalate deposition in the retina has been reported after trauma and in the lens of glaucoma patients [32]. A common theme of many of these disorders is oxidative stress which we believe promotes the generation of more glyoxal and its metabolism to oxalate. This may promote the oxalate deposition described in the aforementioned tissues. This may be impacted by the duration of oxidative stress. For example, end-stage renal disease is associated with oxidative stress. Enoki et al. reported that oxalate deposition in the kidneys of patients with acquired cystic disease was positively correlated with the number of months being on dialysis [29].

Calcium oxalate crystals appear to arise at focal points in most tissues. The underlying changes in oxalate metabolism and the factors associated with this crystal formation warrant further research.

## 7. Summary

We have presented evidence that glyoxal is an important substrate for endogenous oxalate synthesis and as a result may be a critical factor in calcium oxalate urolithiasis. Exogenous sources of glyoxal, such as the diet and the atmosphere, most likely make only a minor contribution. Endogenous sources may be more important and derived from the catabolism of carbohydrates, proteins, and fats through various metabolic reactions, some of which are not yet fully understood. Oxidative stress likely plays a crucial role in glyoxal formation through the interplay between the lipid peroxidation pathway and the autoxidation of sugars and glycated proteins. Calcium oxalate crystal deposition has been noted in tissues under conditions of increased oxidative stress. There is still a great need for further study to identify the associations between glyoxal formation and oxalate synthesis as well as to decipher ways to modify these interactions.
